# Umbilical cord stem cells therapy against bacterial pneumonia based on zebrafish pneumonia model

**DOI:** 10.3389/fphar.2025.1546193

**Published:** 2025-04-09

**Authors:** Xueli Chen, Jian Jin, Xia Chen, Yabin Wu, Youjun Guo, Zuoyu Qian, Hui Huang

**Affiliations:** ^1^ Department of pediatric pulmonology, Maternal and Child Health Hospital of Hubei Province, Wuhan, China; ^2^ Department of Medical Equipment, Wuhan No. 1 Hospital, Wuhan, China; ^3^ Beijing Duan-Dian Pharmaceutical Research and Development Co., Ltd., Beijing, China

**Keywords:** umbilical cord stem cells, bacterial pneumonia, zebrafish pneumonia model, therapeutic effects, inflammatory factors

## Abstract

**Background:**

The increasing incidence and mortality rates of respiratory system diseases globally pose a significant public health challenge. Bacterial pneumonia is one of the leading risk factors for acute lung injury. Conventional antibiotics face inherent limitations, particularly the increase in bacterial resistance and inability to suppress inflammatory states, underscoring the urgent need for novel approaches to combat bacterial pneumonia.

**Methods:**

This study evaluated the therapeutic effects of umbilical cord mesenchymal stem cells (UC-MSCs) on bacterial pneumonia in a zebrafish model, focusing on their impact on macrophage and neutrophil counts and their inhibitory effects on *in vivo* inflammatory responses. The anti-inflammatory mechanisms of UC-MSCs, including their effects on the secretion of inflammatory factors IL-1β, IL-6, and TNF-α, as well as their regulation of NLRP3, TLR4, and NF-kB mRNA expression and NLRP3, p65, and TLR4 protein levels, were further investigated.

**Results:**

Our study found that UC-MSCs can effectively inhibit the development of bacterial pneumonia, primarily by reducing the number of macrophages and neutrophils and inhibiting the secretion of inflammatory factors IL-1β, IL-6, and TNF-α, thereby suppressing *in vivo* inflammatory reactions. Additionally, UC-MSCs significantly downregulated the expression of NLRP3, TLR4, and NF-kB mRNA, as well as the levels of NLRP3,TLR4, and p65 proteins.

**Conclusion:**

UC-MSCs demonstrate promising potential in the treatment of bacterial pneumonia. This study provides important reference for the therapeutic effects and mechanisms of stem cell treatment of bacterial pneumonia, offering new avenues for clinical applications.

## 1 Introduction

The continuous rise in the incidence and mortality rates of respiratory system diseases has turned it into a global public health issue (Yuksel et al., 2023). Acute lung injury (ALI), caused by various direct or indirect damaging factors, leads to injuries of alveolar epithelial cells and capillary endothelial cells, presenting as diffuse interstitial and alveolar edema (Mokra, 2020). This results in acute hypoxic respiratory insufficiency, and in severe cases, can lead to acute respiratory distress syndrome (ARDS).

Pneumonia is one of the most prevalent risk factors for ALI (Kumar, 2020). Pneumonia is a common and potentially severe inflammatory condition of the lungs, characterized by inflammation in the lung parenchyma and alveolar spaces. This inflammation is primarily caused by infections, with bacteria being one of the most significant etiological agents (Kato, 2024). The pathogenesis of pneumonia involves a complex interplay between the invading pathogens and the host immune response, leading to the clinical manifestations observed in patients (Xiao et al., 2025). The specific characteristics of inflammatory infiltration in pneumonia can vary depending on the type of pathogen and the stage of the disease. For example, in bacterial pneumonia, the inflammatory response is typically more robust and can lead to significant consolidation of lung tissue. This process is characterized by the accumulation of immune cells, fluid, and proteins in the alveolar space, which can impair gas exchange and lead to symptoms such as dyspnea and hypoxemia. Lipopolysaccharide (LPS), the main component of the outer membrane of Gram-negative bacteria, has been identified as a crucial risk factor in the pathogenesis of ALI. Inflammatory cells such as neutrophils are recruited and activated, and then enter the lung tissue and release inflammatory mediators when ALI occurs (Long et al., 2022). Macrophages, as important immune cells in the body, are key factors involved in maintaining the homeostasis of the lung microenvironment (Bazaz et al., 2023). Due to their high plasticity, they can polarize into different subtypes under different conditions: classically activated macrophages (M1) and alternatively activated macrophages (M2), which possess pro-inflammatory and anti-inflammatory effects respectively. The M1/M2 phenotype is closely associated with the occurrence and development of lung diseases (Arora et al., 2018). Studying the functional mechanisms of neutrophils and macrophages in lung injury diseases is of great significance and can provide new ideas for exploring the potential of macrophage polarization in regulating lung inflammation. Research has demonstrated that exosomes derived from polymorphonuclear neutrophils stimulated by TNF-α can enhance the activation of M1 macrophages and primed macrophage for pyroptosis (Jiao et al., 2021).

Toll-like receptor 4 (TLR4) is a type of pattern recognition receptor in the innate immune system (Wei et al., 2023). Its activation can trigger a cascade reaction and activate the nuclear factor-kB (NF-kB) that mediates inflammatory responses, playing a vital role in the treatment of acute lung injury. When viruses and bacteria invade the body, they can elicit the innate immune response, trigger the Toll-like receptor family, and subsequently activate the NF-kB signaling pathway and the NLRP3 inflammasome response, increasing the secretion of the inflammatory factor IL-1β through Caspase-1 (Tang et al., 2021). LPS has been found to induce autophagy in murine alveolar macrophages in the context of acute lung injury. This process notably elevates the serum and alveolar lavage fluid concentrations of pro-inflammatory cytokines TNF-α and IL-6, with corresponding severe pathological changes observed in lung tissue (Wang et al., 2023).

With the development of regenerative medicine, cell therapy has emerged as a new strategy for treating lung diseases. Mesenchymal stem cells (MSCs) are multipotent adult stem cells with strong self-renewal and differentiation capabilities. MSCs are highly immunomodulatory and exhibit both anti-inflammatory and pro-trophic properties, which are critical for tissue repair and homeostasis. Their therapeutic effects are largely mediated through the secretion of various factors, including cytokines, chemokines, growth factors, and extracellular vesicles. For example, MSCs can release anti-inflammatory cytokines such as IL-10 and TGF-β, which help suppress excessive immune responses and promote tissue repair (Zhou and Shi, 2023). Additionally, MSC-derived exosomes have been shown to mediate immunoregulatory functions and transfer bioactive molecules to target cells, enhancing their therapeutic potential (Han et al., 2022). MSCs have become ideal seed cells for the treatment of acute and chronic lung diseases due to their biological characteristics, low immunogenicity and abundant sources (Gopalarethinam et al., 2023). A large number of studies have shown that MSCs have been used in the treatment of acute and chronic lung injury diseases such as COVID-19, acute lung injury, acute respiratory distress syndrome, chronic obstructive pulmonary disease, and asthma (Wang et al., 2021). Zebrafish are genetically very similar to humans, and many of the genes associated with human diseases are also expressed and function the same in zebrafish (Tyrkalska et al., 2023). In addition, zebrafish embryos are transparent, and researchers can directly observe the development and pathological processes inside, which has significant advantages in observing macrophages and neutrophils in pneumonia models (Ignatius and Langenau, 2011). Conventional bacterial pneumonia is usually treated with antibiotics, but this treatment method face inherent limitations, particularly the increase in bacterial resistance and inability to suppress inflammatory states. This study aims to explore the therapeutic potential of umbilical cord mesenchymal stem cells (UC-MSCs) in a zebrafish model of bacterial pneumonia.

## 2 Methods

### 2.1 Zebrafish culture condition

Transgenic neutrophil/macrophage green fluorescent zebrafish at 5 days post-fertilization (5 dpf) (Hunter Biotechnology Co., Ltd., Hangzhou, China) were placed in six-well plates at a density of 30 zebrafishper well at 28°C (Aceto et al., 2016). In control group, zebrafish did not receive any special treatment. In model control group, zebrafish were injected with LPS (100 ng per zebrafish) into the swim bladder to establish a bacterial pneumonia model. In positive control group, dexamethasone acetate (100 μM) was used to treat bacterial pneumonia zebrazebrafish. In experimental group, UC-MSCs (purchased from Shenzhen Mosel Biomedical Technology Development Co., Ltd., with a valid ethical approval certificate, and were used at the P5 passage) (62.5, 125, 250, 500, 1,000 cells per zebrafish) were injected into the swim bladder of the bacterial pneumonia zebrafish. The relevant parameters were detected 24 h after injection of UC-MSCs. The study was approved by the Institutional Animal Care and Use Committee (IACUC-2024-9283-01).

### 2.2 Evaluation of neutrophil change

The neutrophil number was one of the indicator to evaluate the efficacy of the UC-MSCs in anti-bacterial pneumonia (Domon and Terao, 2023). After 24 h of treatment for bacterial pneumonia zebrafish at 28°C, 10 zebrafish were randomly selected from each group and photographed under a fluorescence microscope. The morphology of neutrophils is clear and the particles are distinct, so the number of neutrophils can be counted directly by fluorescence images. The data were collected using NIS-Elements D 3.20 advanced image processing software, and the number of neutrophils in the swim bladder of zebrafish was analyzed.

### 2.3 Evaluation of macrophage change

After 24 h of treatment at 28°C, 10 zebrafish were randomly selected from each group and photographed under a fluorescence microscope. The data were collected and analyzed using ImageJ software, and the fluorescence intensity of macrophages in the zebrafish swim bladder was analyzed. Macrophage particles are not clearly displayed, have no specific shape, and are not easy to count, so the fluorescence intensity is used to count the number of cells. The statistical analysis of this indicator was used to evaluate the anti-bacterial pneumonia efficacy of the UC-MSCs.

### 2.4 Histopathology analysis

The zebrafish were fixed with 4% tissue fixative (Solarbio Technology Co., Ltd., Beijing, China), and after the dehydration, embedding, sectioning, and staining steps, they were subjected to histological H&E staining analysis. The anti-bacterial pneumonia efficacy of the sample was evaluated through tissue pathological analysis of the swim bladder.

### 2.5 Inflammatory factor measurement

Zebrafish tissue homogenate was obtained, and the supernatant was obtained by centrifugation at 3,000 rpm for 15 min. The IL-1β, IL-6, and TNF-a were measured by IL-1β, IL-6, and TNF-a assay kit (Meimian Biotechnology Co., Ltd., Jiangsu, China), respectively.

### 2.6 Real-time quantitative PCR

Quantitative reverse transcription polymerase chain reaction (qRT-PCR) was conducted to compare gene expression levels among the groups. Total mRNA was extracted from the Zebrafish using TRIzol Reagent (Tiangen Biochemical Technology Co., Ltd., Beijing, China). The RevertAid First Strand cDNA Synthesis Kit (Tsingke Biotechnology, China) was used to synthesize cDNA from total RNA samples. RT-PCR reactions were run on an ABI StepOnePlus™ (Applied Biosystems) (Zhu et al., 2023). The primers were showed as follows: TLR4-forward, 5-TGC GTG GAG GTG GTT CCT AAT A-3 and reverse, 5-CTT GGT TGA GAA GGG GAG GTT G-3; NLRP3-forward, 5-ATT ACC CGC CCG AGA AAG G-3 and reverse, 5-CAT GAG TGT GGC TAG ATC CAA G-3; NF-kB-forward, 5-TGA CGG GAG GGG AAG AAA TC-3 and reverse, 5-TGA ACA AAC ACG GAA GCT GG-3; GAPDH-forward, 5-GAA GGT GAA GGT CGG AGT C-3 and reverse, 5-GAA GAT GGT GAT GGG ATT TC-3.

### 2.7 Western blot analysis

The zebrafish cells were lysed using a RIPA lysis buffer (Solarbio Technology Co., Ltd., Beijing, China). The proteins were subjected to immunoblot analysis with indicated antibodies. The target antibodies included NLRP3 (68102-1-Ig, Proteintech Co., Ltd., Hubei, China), p65 (10745-1-AP, Cell Signaling Technologies, United States), TLR4 (19811-1-AP, Cell Signaling Technologies, United States), K48-linkage Specific Polyubiquitin (D9D5).

### 2.8 Statistical analysis

The statistical processing results were expressed as mean ± SE. The paired t-test was used for comparison between the two groups. The statistical analysis was performed using SPSS 26.0 software, and *p < 0.05, **p < 0.01, ***p < 0.001 indicated a statistically significant difference. Three replicate experiments were conducted to meet the statistical requirements.

## 3 Results

### 3.1 Effect of UC-MSCs treatment on zebrafish phenotype and death rate

This study was based on zebrafish model to investigate the mechanism of UC-MSCs in the treatment of bacterial pneumonia (Yang et al., 2017). In general, the treatment is based on the fact that it does not produce strong side effects, and if the method causes the death of the individual, it will do more harm than good. Therefore, this study first evaluated the effect of different doses of UC-MSCs on the survival rate of zebrafish. In this study, zebrafish were induced to form bacterial pneumonia by injection of LPS into the swim bladder of zebrafish, followed by injection of UC-MSCs to treat the disease. The survival rate and phenotype of zebrafish were observed 24 h later to confirm that the therapeutic dose of UC-MSCs used in the study did not produce serious adverse effects. The results showed that the zebrafish without LPS injection (the control group) exhibited no mortality and no obvious abnormal phenotypes during the experiments. The model control group injected with LPS to induce bacterial pneumonia also did not observe the death of zebrafish, and the phenotype was not obvious abnormal, providing a stable baseline for further comparative analysis. In experimental group, injection of 62.5, 125, 250, 500, 1000 UC-MSCs per zebrafish did not cause zebrafish death, and the phenotype was similar to the model control group (Table 1). Therefore, subsequent studies of UC-MSCs for the treatment of bacterial pneumonia use this dose range.

**TABLE 1:** Effect of UC-MSCs treatment on zebrafish phenotype and death rate (n = 30).

Group	Mesenchymal stem cells per zebrafish	Number of dead zebrafish	Death rate (%)	Phenotype
Control group	—	0	0	No obvious abnormality
Model control group	—	0	0	No obvious abnormality
Experimental group	62.5	0	0	Similar to the model control group
	125	0	0	Similar to the model control group
	250	0	0	Similar to the model control group
	500	0	0	Similar to the model control group
	1,000	0	0	Similar to the model control group

### 3.2 Evaluation of anti-bacterial pneumonia efficacy by neutrophil change

**FIGURE 1:**
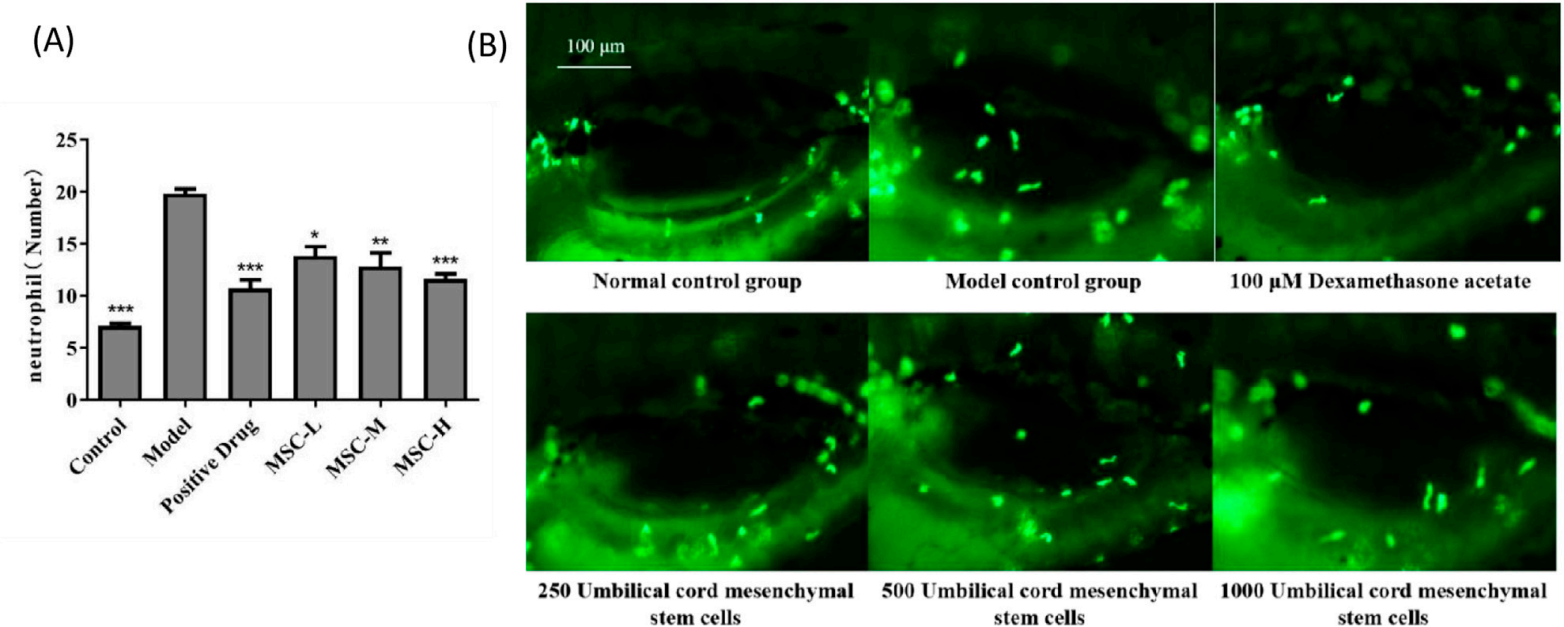
The number of neutrophils in the swim bladder of zebrafish after UC-MSCs treatment. **(A)** The number of neutrophils in the swim bladder of zebrafish in each group; **(B)** Fluorescent images of neutrophils in the swim bladder of zebrafish in each group. Note: the green fluorescent particles represent neutrophils. *p < 0.05, **p < 0.01, ***p < 0.001 compared to the model control group.

In this study, zebrafish bacterial pneumonia model was established by injecting LPS. LPS, a major component of the outer membrane of Gram-negative bacteria, has been identified as a key risk factor in the pathogenesis of acute lung injury, and its use in animal models to induce acute lung injury has been widely reported. During LPS-induced acute lung injury, inflammatory cells such as neutrophils are recruited and activated. After activation, inflammatory cells penetrate into lung tissue. Because zebrafish are transparent, this study uses fluorescent labeling method to visually see the distribution of neutrophils, which greatly avoids the use of conventional flow cytometry and other experimental methods, and greatly improves the experimental efficiency. The results showed that the number of neutrophils in the model control group was significantly higher than that in the control group, which was consistent with the characteristics of bacterial pneumonia. Compared with the model control group, the number of neutrophils decreased significantly after dexamethasone administration in the positive control group. The number of neutrophils in zebrafish swim bladder also decreased significantly after injection of UC-MSCs in the experimental group, indicating that UC-MSCs have anti-bacterial pneumonia effect. In addition, the greater the number of UC-MSCs injected, the more effective the treatment of bacterial pneumonia (Figure 1).

### 3.3 Evaluation of anti-bacterial pneumonia efficacy by fluorescence intensity of macrophages

**FIGURE 2:**
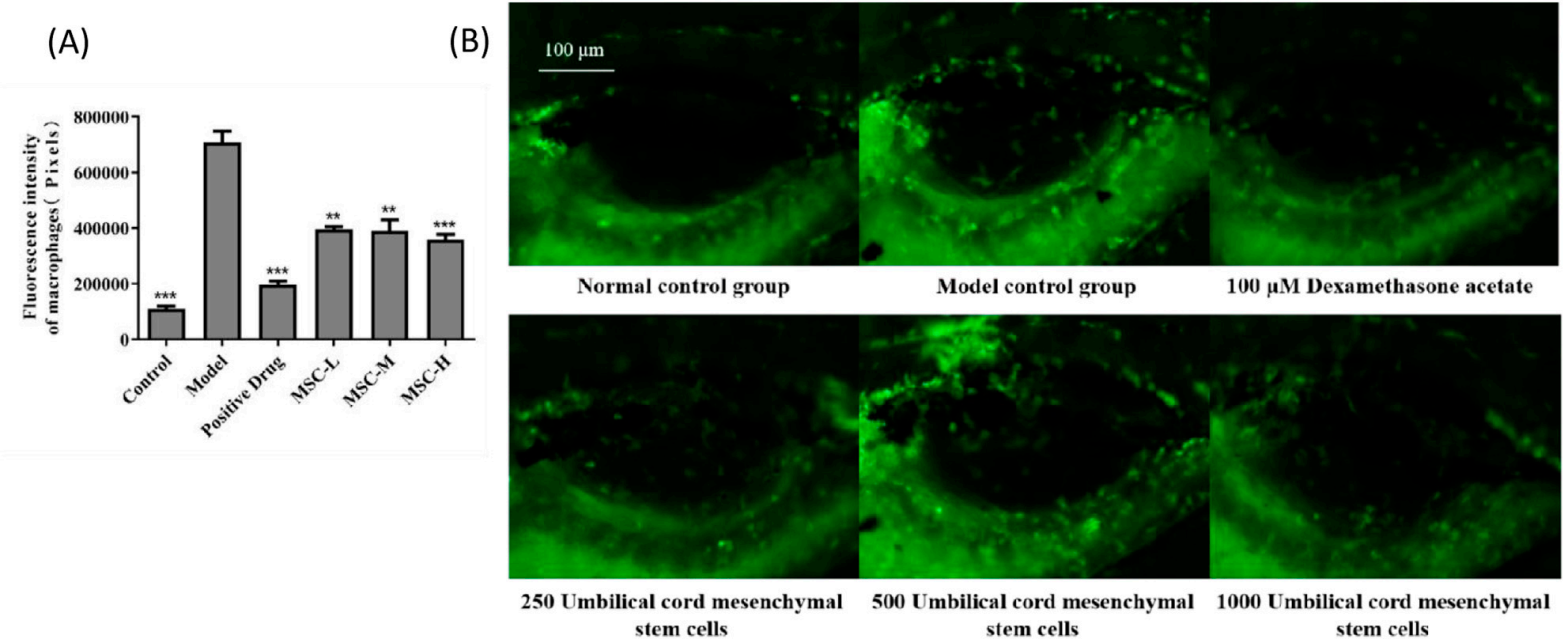
The fluorescence intensity of macrophages in the swim bladder of zebrafish after UC-MSCs treatment. **(A)** Fluorescence intensity of macrophages in the swim bladder of zebrafish in each group. **(B)** Fluorescent images of macrophages in the swim bladder of zebrafish in different groups. Note: the green fluorescent particles represent macrophages. *p < 0.05, **p < 0.01, ***p < 0.001 compared to the model control group.

In this study, macrophages in zebrafish were labeled with green fluorescence, and the distribution of macrophages could be visually observed. The anti-bacterial pneumonia efficacy of UC-MSCs was analyzed by observing the fluorescence intensity of macrophages in swim bladder. The experimental results showed that the fluorescence intensity of macrophages in the model control group was significantly higher than that in the control group, which was consistent with the characteristics of pulmonary inflammation. Compared with the model control group, the fluorescence intensity of macrophages in the positive control group was significantly reduced after dexamethasone administration. The fluorescence intensity of macrophages in zebrafish swim bladder also decreased significantly after injection of UC-MSCs in the experimental group, indicating that UC-MSCs have anti-bacterial pneumonia effect. In addition, the more UC-MSCs were injected, the more effective they were in treating bacterial pneumonia (Figure 2).

### 3.4 Histopathology of swim bladder after treatment

**FIGURE 3:**
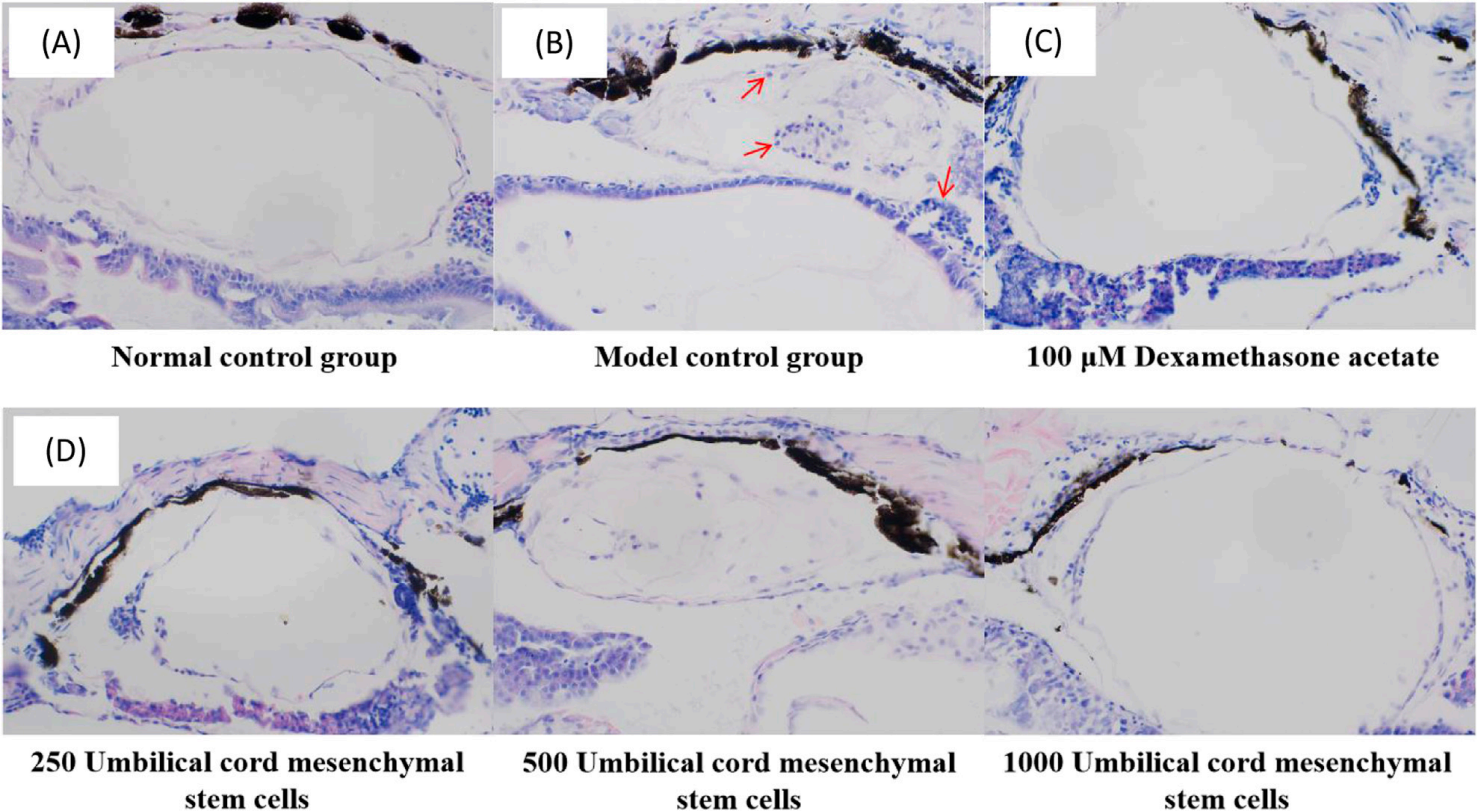
Histopathological images of zebrafish swim bladder in **(A)** control group, **(B)** model control group, **(C)** positive control group and **(D)** experimental group. The red arrow points to inflammatory cells.

Under the experimental conditions, the zebrafish in the control group had regular bladder tissue structure and intact cell morphology, with uniform cytoplasm. The model control group had obvious inflammatory infiltration within the zebrafish bladder, indicating that the bacterial pneumonia model of zebrafish was successfully established. In the positive control group, the inflammatory infiltration of zebrafish was reduced after administration of dexamethasone acetate, indicating that dexamethasone acetate had an therapeutic effect on LPS induced bacterial pneumonia. In the experimental group, the inflammatory infiltration in the swim bladder of zebrafish injected with UC-MSCs was significantly reduced, and the tissue structure of swim bladder was similar to that of control group. The results showed that UC-MSCs had anti-bacterial pneumonia efficacy, which was manifested by reduced inflammatory infiltration of swim bladder tissue (Figure 3).

### 3.5 The UC-MSCs therapy reduces inflammatory factors

**FIGURE 4:**
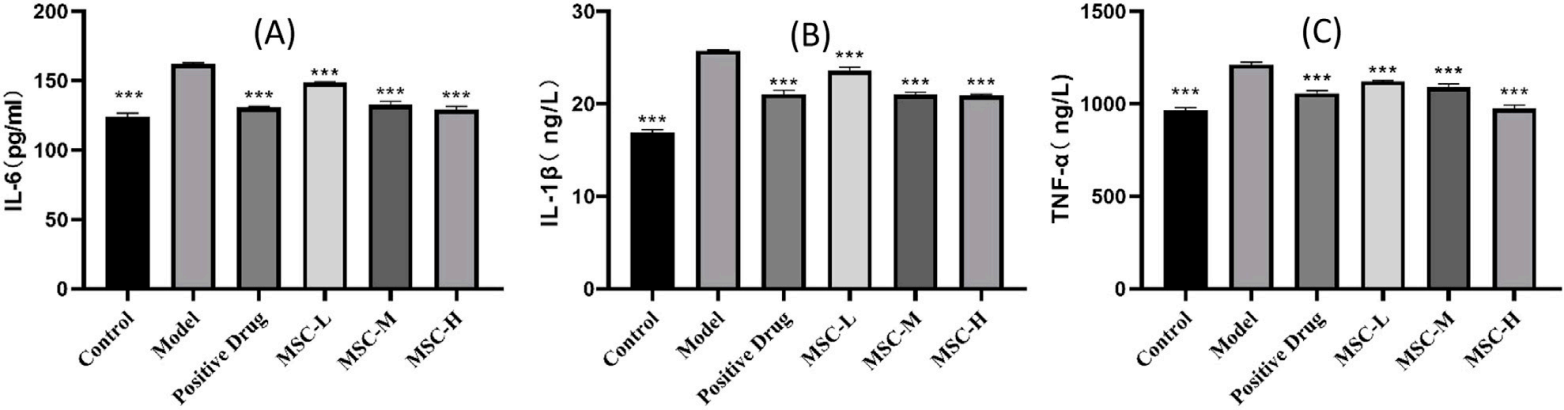
Contents of inflammatory factors in control group, model control group, positive control group and experimental group. **(A)** IL-6 content in each group. **(B)** IL-1β content in each group. **(C)** TNF-a content in each group. **p < 0.01, ***p < 0.001 compared with the model control group.

The three indicators of IL-1β, IL-6 and TNF-a are the research hotspots related to inflammation indicators (Schultheiß et al., 2022). In this study, the results showed that IL-1β, IL-6 and TNF-a in control group were lower than those of the model control group, and there were extremely significant statistical differences (p < 0.001), indicating that LPS-induced zebrafish pneumonia models elevated inflammatory factors. Compared with the model control group, the contents of IL-1β, IL-6 and TNF-a in all experimental groups were lower than those in the model group, and there were extremely significant statistical differences (p < 0.001). This study showed that the injection of UC-MSCs in the treatment of bacterial pneumonia has the effect of reducing inflammatory factors (Figure 4).

### 3.6 The UC-MSCs therapy inhibits TLR4/NF-kB/NLRP3 signaling pathway

**FIGURE 5:**
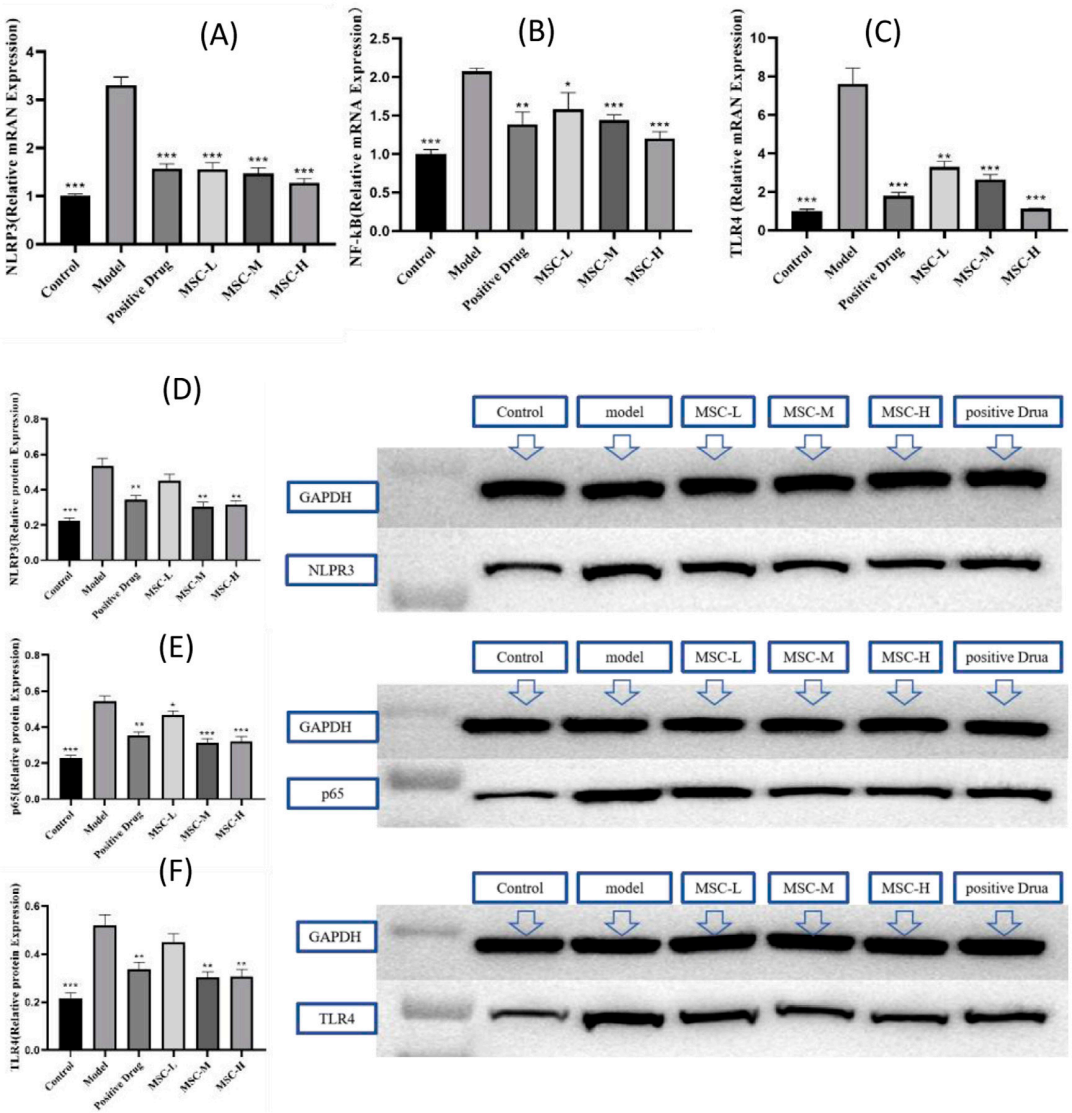
The UC-MSCs therapy inhibits TLR4/NF-kB/NLRP3 signaling pathway. The mRNA expression of **(A)** NLRP3, **(B)** NF-kB, **(C)** TLR4; the protein expression of **(D)** NLRP3, **(E)** p65 and **(F)** TLR4. **p < 0.01, ***p < 0.001 compared with the model control group.

Studies have shown that when bacteria invade the body, they can cause the innate immune response, trigger the toll receptor family, and then activate the NF-kB signaling pathway and activate the NLRP3 inflammatome response. The PCR results of this study showed that compared with the normal group, the expression levels of NLRP3, NF-kB and TLR4 genes in the model control group were higher than those in the normal group. Compared with the model control group, NLRP3, NF-kB and TLR4 gene expressions in all experimental groups were smaller than those in the model group. The expressions of NLRP3, NF-kB and TLR4 genes all showed a downward trend with the increase of the injection dose of UC-MSCs (Figures 5A–C). The results of WB experiment showed that the expression of NF-kB p65 protein in zebrafish injected with UC-MSCs at a low dose (250/tail) was slightly lower than that in the model group, while NLRP3 and TLR4 were not significantly changed. The protein expressions of NLRP3, p65 and TLR4 of zebrafish injected with UC-MSCs were lower than those of the model group (Figures 5D–F). In conclusion, the treatment of bacterial pneumonia by UC-MSCs can be achieved by down-regulating the expression of NLRP3, TLR4 and NF-kB mRNA and protein.

## 4 Discussion

Our study found that UC-MSCs can effectively inhibit the development of bacterial pneumonia, primarily by reducing the number of macrophages and neutrophils and inhibiting the secretion of inflammatory factors IL-1β, IL-6, and TNF-α, thereby suppressing *in vivo* inflammatory reactions. Additionally, UC-MSCs significantly downregulated the expression of TLR4, NF-kB and NLRP3 mRNA, as well as the levels of TLR4, p65 and NLRP3 proteins, suggesting UC-MSCs may potentially mediate their therapeutic effects via blocking the TLR4/NF-κB/NLRP3 pathway.

MSCs, alternately referred to as mesenchymal stromal cells or medicinal signaling cells, have demonstrated the ability to regulate excessive immune responses and severe inflammation, facilitate tissue regeneration, and produce antimicrobial agents (Mishra et al., 2020). With a proven safety record via intravenous administration, these cells are under investigation for treating a range of conditions, including autoimmune diseases, systemic lupus erythematosus, various inflammatory diseases. Studies have indicated that MSCs can reduce pulmonary inflammation and fibrosis and show promising, albeit variable, outcomes in the treatment of ARDS of both viral and nonviral origins (Yip et al., 2020). MSCs can be derived and expanded from several tissues, with umbilical cord (UC) being a notable source. UC-MSCs are partic ularly favored in cellular therapy trials, including those for COVID-19 (Lanzoni et al., 2021). Studies have shown that delayed MSCs therapy enhanced resolution of lung injury induced by antibiotic resistant *Klebsiella* infection and favorably modulated immune cell profile (Byrnes et al., 2023). MSCs derived conditioned medium is a potent antimicrobial agent and is compatible with vibrating mesh nebulization delivery (McCarthy et al., 2020). Consistent with these research findings, our results also demonstrate the efficacy of UC-MCSs in treating bacterial pneumonia. Our research has validated this conclusion at the animal level as a whole. Animal models play a crucial role in deciphering the mechanisms behind numerous human diseases and in identifying potential therapeutic targets. These models are instrumental for evaluating the effectiveness and safety of treatments, determining optimal administration methods, and elucidating the pharmacokinetic and pharmacodynamic properties of drugs. This study selected zebrafish as the model for bacterial pneumonia because zebrafish has the following advantages: low maintenance cost, short lifespan, high fecundity levels, high throughput, ease of manipulation, high homology of genes with human genome (Tyrkalska et al., 2023). In the present study, the model control group had obvious inflammatory infiltration within the zebrafish bladder, indicating that the bacterial pneumonia model of zebrafish was successfully established, which is consistent with previous studies (Saralahti et al., 2023). This study established a zebrafish model of bacterial pneumonia using LPS, a well-established method to induce acute lung injury and mimic the inflammatory response seen in human bacterial pneumonia (Li et al., 2021). The use of LPS in our model allowed us to investigate the recruitment and activation of neutrophils, a key aspect of the immune response in pneumonia. To observe the anti-inflammatory effect and mechanism of MSCs in zebrafish model provides an important theoretical basis for the development of new therapeutic methods for bacterial pneumonia.

Our findings show a significant increase in neutrophil numbers in the model control group, validating the model’s ability to replicate the inflammatory characteristics of bacterial pneumonia. The transparency of zebrafish enabled us to use a fluorescent labeling method to visualize neutrophil distribution, offering a non-invasive and efficient alternative to traditional methods like flow cytometry. This approach not only simplifies the experimental process but also enhances the accuracy of monitoring inflammation in real-time. Our results demonstrate that dexamethasone, a corticosteroid with known anti-inflammatory properties, significantly reduced neutrophil and macrophage fluorescence intensity in the positive control group, confirming its efficacy in mitigating LPS-induced inflammation. Similarly, the experimental group treated with UC-MSCs showed a significant decrease in neutrophil and macrophage numbers in the zebrafish swim bladder, indicating that UC-MSCs possess an anti-inflammatory effect that could be beneficial in treating bacterial pneumonia. Furthermore, the study observed a correlation between the number of UC-MSCs injected and the effectiveness of bacterial pneumonia treatment, suggesting a dose-dependent therapeutic potential for UC-MSCs. This finding is particularly significant as it implies that the therapeutic effects of UC-MSCs can be optimized by adjusting the dosage. An analysis of COVID-19 case series data from PubMed literature disclosed significant variability in the therapeutic dosing of UC-MSCs (Hsueh et al., 2023).

The cytokines IL-1β, IL-6, and TNF-α, which are key indicators of inflammation (Schultheiß et al., 2022), were found to be elevated in the model control group compared to the control group, with extremely significant statistical differences, confirming the inflammatory nature of the LPS-induced pneumonia model. A comprehensive analysis has assessed the efficacy of stem cells in managing systemic inflammation resulting from burns within animal models (Eldaly et al., 2022). The review suggests that MSCs could potentially reduce inflammation caused by burns by lowering the levels of inflammatory cytokines in the serum (Eldaly et al., 2022). Similarly, our study found that all experimental groups treated with UC-MSCs exhibited lower levels of these cytokines than the model control group, indicating a potent anti-inflammatory effect of UC-MSCs *in vivo*.

The innate immune response, triggered by bacterial invasion, involves the activation of toll-like receptors (TLRs), which in turn activate the NF-kB signaling pathway and the NLRP3 inflammasome response (Zhang et al., 2021). Rg1 may alleviate LPS induced inflammation and apoptosis in NRCMs and septic mice by blocking the TLR4/NF-κB/NLRP3 pathway (Luo et al., 2020). Our PCR results corroborate previous studies by showing increased expression of NLRP3, NF-kB, and TLR4 in the model control group. Strikingly, all experimental groups treated with UC-MSCs showed a reduction in the expression of these genes. Furthermore, the expression of NLRP3, NF-kB, and TLR4 genes displayed a downward trend with increasing doses of UC-MSCs, indicating a dose-dependent effect. Western blot experiments revealed that while low-dose UC-MSC treatment slightly reduced NF-kB p65 protein expression, higher doses led to a more pronounced decrease in the protein expressions of NLRP3, p65, and TLR4. These findings suggest that UC-MSCs can exert their therapeutic effects by downregulating the expression of key inflammatory mediators at both the mRNA and protein levels.

There are certain limitations associated with this study. The zebrafish model, while valuable for initial screening and mechanistic studies, may not fully replicate the complexity of the human immune response. Future studies should aim to validate our findings in preclinical models and ultimately in clinical trials to assess the safety and efficacy of UC-MSCs in treating bacterial pneumonia. The study revealed a notable reduction in neutrophil and macrophage counts in the swim bladder of zebrafish with bacterial pneumonia following treatment with UC-MSCs; however, the interplay between these two cell populations remains to be further elucidated.

In conclusion, our study underscores the potential of UC-MSCs as a novel therapeutic strategy for bacterial pneumonia. The immunomodulatory and anti-inflammatory properties of UC-MSCs, as demonstrated in our research, warrant further investigation for their clinical application in managing pulmonary inflammation and other inflammatory diseases.

## Data Availability

The original contributions presented in the study are included in the article, further inquiries can be directed to the corresponding author.
